# Long-lost friend is back in the game

**DOI:** 10.1016/j.jlr.2021.100072

**Published:** 2021-04-02

**Authors:** Sander Kersten

**Affiliations:** Nutrition, Metabolism and Genomics Group, Division of Human Nutrition and Health, Wageningen University, Wageningen, The Netherlands

**Keywords:** ANGPTL, angiopoietin-like protein family

Triglycerides (TGs) are transported in the bloodstream by TG-rich lipoproteins in the form of chylomicrons and VLDLs. The hydrolysis of circulating TGs is rate-limiting for their uptake into tissues and is catalyzed by the enzyme lipoprotein lipase (LPL) (1). The activity of LPL in different tissues is extensively regulated to be able to adjust to changes in lipid availability and demand. The regulation of LPL is mainly carried out at the posttranslational level and is mediated by two groups of proteins. The first group consists of the apolipoproteins C1, C2, C3, E, and A5, while the second group is composed of three members of the angiopoietin-like protein family (ANGPTL): ANGPTL3, ANGPTL4, and ANGPTL8 (1).

Of this list of proteins, the most elusive LPL regulator is probably APOA5. APOA5 was discovered independently by two groups in 2001. Pennacchio *et al*. (2) identified APOA5 by comparative sequencing of the mouse and human *APOAI*/*CIII*/*AIV* gene cluster, while van der Vliet identified *APOA5* as the most highly induced gene after partial hepatectomy (3). Targeted inactivation or overexpression of APOA5 in mice clearly demonstrated the marked suppressive effect of APOA5 on plasma TG concentrations (2, 4). The role of APOA5 in human TG metabolism is strongly supported by human genetics studies, which have consistently found that common *APOA5* variants are associated with elevated plasma TG levels (5).

How APOA5 lowers plasma TGs has remained controversial. A report suggested that APOA5 reduces VLDL-TG production (6). Alternatively, APOA5 may promote the receptor-mediated removal of remnants of TG-rich lipoproteins (7, 8). The most compelling data indicate that APOA5 stimulates LPL-mediated lipolysis of TG-rich lipoproteins (6, 7, 9). To many scientists in the field, however, this mechanism has not been completely satisfactory. APOA5 is present in plasma at concentrations several orders of magnitude lower than that of other apolipoproteins. People have not been able to get their head around how an apolipoprotein that is present in plasma at such low concentrations would be able to regulate LPL.

Here, inspiration may be taken from the angiopoietin-like proteins ANGPTL3, ANGPTL4, and ANGPTL8. These proteins function as inhibitors of LPL and are present in plasma at concentrations that resemble the plasma APOA5 concentration. Although ANGPTLs were initially believed to operate individually, recent findings indicate that ANGPTL3 and ANGPTL4 form complexes with ANGPTL8 (10, 11, 12). Importantly, the ANGPTL3/ANGPTL8 complex is a much more potent LPL inhibitor than either ANGPTL3 or ANGPTL8 alone (13). As an exciting new revelation, Chen *et al*. (14) now find that APOA5 binds to the ANGPTL3/ANGPTL8 complex. Through this mechanism, APOA5 interferes with LPL inhibition by ANGPTL3/ANGPTL8.

Chen *et al.* stumbled upon APOA5 when trying to identify proteins in human serum that associate with immobilized ANGPTL3/8. They also succeeded in immunoprecipitating APOA5 from human serum when using an antibody directed against the ANGPTL3/ANGPTL8 complex. Subsequent biolayer interferometry experiments showed that recombinant APOA5 is able to efficiently bind to the ANGPTL3/8 complex. At the functional level, recombinant APOA5 impaired the ability of the ANGPTL3/ANGPTL8 complex to inhibit LPL. By contrast, APOA5 did not influence LPL inhibition by ANGPTL3 and ANGPTL4 alone or in the absence of any angiopoietin-like proteins. Answering to the original conundrum about the low plasma APOA5 concentration, it was found that the suppression of ANGPTL3/8-mediated LPL inhibition by APOA5 occurred at a molar ratio consistent with the plasma concentrations of APOA5 and ANGPTL3/8.

After 15 years of uncertainty, these new biochemical data represent a potential breakthrough in our molecular understanding of how APOA5 lowers plasma TGs. Whether APOA5 also interferes with the function of ANGPTL3/ANGPTL8 in vivo is still unknown. Accordingly, it would be of interest to investigate the effect of overexpression of APOA5 or injection of recombinant APOA5 on postprandial plasma TGs or on plasma TG clearance in mice deficient in ANGPTL3 or ANGPTL8.

This exhilarating new discovery might create potential novel opportunities for the therapeutic targeting of hyperlipidemia, an important risk factor for atherosclerotic cardiovascular disease. Currently, ANGPTL3 is a hot target for the treatment of atherosclerotic cardiovascular disease. Inactivation of ANGPTL3 via antisense oligonucleotides and monoclonal antibodies effectively lowers plasma TGs and LDL-C in mouse models and in human volunteers (15, 16). Based on the results presented by Chen *et al*., another therapeutic approach could be to mimic the binding of APOA5 to ANGPTL3/ANGPTL8, thereby relieving LPL inhibition by ANGPTL3/ANGPTL8. To enable the design of such a strategy, better insight is needed into the specific domains in APOA5 and ANGPTL3/ANGPTL8 involved in their mutual interaction.

ANGPTLs and APOA5 have followed a very different path since they were first cloned about twenty years. Although the importance of APOA5 in regulating plasma TG levels quickly gained ground, the recognition of the role of ANGPTLs in controlling human plasma lipoproteins took a much more gradual course. Their mutual interaction adds an exciting new twist to a now rapidly evolving field.
